# Prevalence of diabetes complications in people with type 2 diabetes mellitus and its association with baseline characteristics in the multinational A_1_chieve study

**DOI:** 10.1186/1758-5996-5-57

**Published:** 2013-10-24

**Authors:** Leon Litwak, Su-Yen Goh, Zanariah Hussein, Rachid Malek, Vinay Prusty, Mohammad E Khamseh

**Affiliations:** 1 Endocrinology and Nuclear Medicine Unit, Diabetes and Metabolism Section, Hospital Italiano de Buenos Aires, Buenos Aires, Argentina; 2 Department of Endocrinology, Singapore General Hospital, Singapore; 3 Medical Department, Hospital Putrajaya, Putrajaya, Malaysia; 4 Department of Internal Medicine, CHU Sétif, Sétif, Algeria; 5 Novo Nordisk A/S, Zürich, Switzerland; 6 Endocrine Research Centre (Firouzgar), Institute of Endocrinology & Metabolism, Tehran University of Medical Sciences, Tehran, Iran

**Keywords:** A_1_chieve study, Macro- and microvascular complications, Type 2 diabetes, Insulin analogues

## Abstract

**Background:**

Current International Diabetes Federation guidelines recommend a target HbA_1c_ <7.0%, but many people with diabetes worldwide find this difficult to achieve, increasing their risk of developing complications. This publication examines the prevalence of diabetes complications and its association with baseline characteristics in people with type 2 diabetes who participated in the A_1_chieve study.

**Methods:**

A_1_chieve was a 24-week, multinational, open-label, observational study of 66,726 people with type 2 diabetes who had begun using biphasic insulin aspart 30, insulin aspart, or insulin detemir in routine clinical care. Participants were enrolled from 28 countries across four continents (Asia, Africa, Europe and South America). Baseline measurements of disease characteristics included: glycated haemoglobin (HbA_1c_), fasting (FPG) and post-prandial plasma glucose (PPG), high- and low-density lipoprotein cholesterol (H- or LDL-C), systolic blood pressure (SBP), and body mass index (BMI). Data on complications and use of vascular disease preventative drugs were collected.

**Results:**

Complication rates were high (27.2% had macrovascular complications and 53.5% had microvascular complications), particularly in Russia, and use of vascular disease preventative drugs was lower than expected. Age, BMI, diabetes duration, LDL-C, and SBP were positively associated, and HDL-C negatively associated, with macro- and microvascular complications (all p < 0.05). HbA_1c_ and FPG were negatively associated with macrovascular complications (both p < 0.05), which may be linked to the cross-sectional study design.

**Conclusions:**

These results suggest a worldwide failure to achieve glycaemic targets. Better diabetes management with earlier initiation and optimisation of insulin regimens (e.g., with insulin analogues in the A_1_chieve population) may reduce the prevalence of vascular complications, improve the lives of people with diabetes and reduce the burden on healthcare systems.

## Introduction

Effective management of diabetes requires sustained glycaemic control over many years to lower the risk of macro- and microvascular complications in people with diabetes. The UK Prospective Diabetes Study (UKPDS) found that every 1% reduction in glycated haemoglobin (HbA_1c_) was associated with a 37% decrease in microvascular disease and a 14% reduction in myocardial infarction (MI) [1]. Results of a 10-year follow-up study found that people with type 2 diabetes who maintain good glycaemic control experience benefits many years later, including lower rates of MI and diabetes-related death [2].

Accordingly, International Diabetes Federation (IDF) and joint American Diabetes Association (ADA)/European Association for the Study of Diabetes (EASD) guidance recommend a target HbA_1c_ of <7.0% [3,4]. These guidelines highlight the need to review and to modify therapy regimens when HbA_1c_ goals can no longer be maintained [4,5], which may include initiation and intensification of insulin therapy. Current diabetes guidelines also emphasise that cardiovascular disease risk reduction should be a focus of therapy [4-6]. However, most people with diabetes find it difficult to achieve HbA_1c_ levels <7.0% [7-10], with <40% of people with diabetes in a USA national survey reaching this target [9]. Moreover, in developing regions, including Eastern Europe, Asia, Latin America, and Africa, it has been shown that 22% and 36% of people with type 1 diabetes and type 2 diabetes, respectively, have never had their HbA_1c_ measured, and of those with available HbA_1c_ values, only 25% of people with type 1 diabetes and 36% of people with type 2 diabetes had HbA_1c_ levels <7.0% [11]. This may increase the likelihood of progression of diabetes complications and of developing complications in later life. Reasons for the difficulty in achieving target HbA_1c_ levels may include problems adhering to complex oral and injectable therapies, concerns about insulin tolerability, psychological insulin resistance, fears about the risk of hypoglycaemia, and weight gain after insulin initiation [12-16].

More data are needed to evaluate glycaemic control and guideline adherence by physicians in real-life clinical practice in different geographical regions. These data can be used to assess whether glycaemic control, or any other factors, predict the establishment of diabetes-related complications in various ethnic groups.

A_1_chieve was a multinational, open-label, observational study of people with type 2 diabetes to assess clinical safety and effectiveness of biphasic insulin aspart 30 (NovoMix® 30), insulin aspart (NovoRapid®), or insulin detemir (Levemir®) with or without oral glucose-lowering drugs (OGLDs) in routine clinical care [17]. Participants were enrolled from 28 countries in Asia, Africa, South America and Europe [17]. In this report, diabetes complications data at baseline were assessed by geographical region and the relationship of these complications to other variables (e.g., patient and disease characteristics) was assessed by correlation analyses.

## Methods

This was a 24-week international prospective, multi-centre, open-label, non-interventional study in people with type 2 diabetes mellitus who had started biphasic insulin aspart 30, basal insulin detemir or bolus insulin aspart. These study drugs could be given alone or in combination with other medications that the participants were already taking. Baseline data, before participants received any of these insulin analogues or within 4 weeks of receiving insulin analogues, are reported in this analysis. Participants were evaluated by general physicians or diabetes specialists at the baseline visit, an interim visit approximately 12 weeks after the baseline visit, and a final visit approximately 24 weeks after the baseline visit.

The participants were enrolled in the study between January 2009 and June 2010. The 28 countries were grouped into seven geographical regions: China; South Asia (Bangladesh, India, and Pakistan); East Asia (Indonesia, Korea, Malaysia, Philippines, Singapore, and Taiwan); North Africa (Algeria, Morocco, Tunisia, and Libya); Middle East/Gulf (Egypt, Iran, Jordan, Turkey, Bahrain, Kuwait, Oman, Qatar, Saudi Arabia, United Arab Emirates, and Yemen); South America (Argentina and Mexico); and Europe (Russia).

Inclusion and exclusion criteria were kept to a minimum in order to reflect routine clinical practice as closely as possible. Selection of patients and choice of insulin regimen were at the discretion of the physician based on their clinical judgement. Exclusion criteria were: patients treated with biphasic insulin aspart 30, insulin detemir or insulin aspart (alone or in combination) for more than 4 weeks before enrolment in the study; patients previously enrolled in the study; patients with hypersensitivity to biphasic insulin aspart 30, insulin detemir or insulin aspart or to any of its excipients; and women who were pregnant, breast-feeding or had the intention of becoming pregnant within the next six months.

All local requirements for Health Authorities and Ethics Committee approvals were obtained, if applicable. The study was conducted in accordance with the Declaration of Helsinki [18] and the Guidelines for Good Pharmacoepidemiology Practice [19].

### Assessments

Only baseline measurements are reported in this publication, which included patient characteristics, disease characteristics (metabolic control), diabetic macro- and microvascular complications, and use of vascular disease preventative drugs. These data were tabulated by total study population and by geographical region.

Patient characteristics assessed at baseline included: age, sex, diabetes duration, and body mass index (BMI). Disease-related characteristics assessed at baseline included: HbA_1c_, fasting plasma glucose (FPG) before breakfast, post-prandial plasma glucose (PPG) after breakfast, total cholesterol (TC), triglycerides (TG), creatinine, high-density lipoprotein cholesterol (HDL-C), low-density lipoprotein cholesterol (LDL-C), systolic blood pressure (SBP), and quality of life (QoL). QoL was assessed using the standardised EQ-5D™ where 100 = best imaginable health and 0 = worst imaginable health [20].

Since this is an open label, observational study, all complications data were collected from available medical records or memory, with no additional clinical assessments performed. Data were collected for the following macrovascular complications based on the physician’s clinical judgement: MI, angina, peripheral vascular disease, stroke, heart failure, atrial fibrillation, and left ventricular hypertrophy. Data were collected for the following microvascular complications: renal disease (microalbuminuria, gross proteinuria, end-stage renal disease), eye problems (background diabetic retinopathy, proliferative diabetic retinopathy, severe vision loss, macular oedema, and cataract), foot ulcer (uninfected ulcer, infected ulcer, healed ulcer, and history of amputation), and diabetic neuropathy. Data collected on use of vascular disease preventative drugs included use of renin-angiotensin system (RAS) blockers, aspirin and statins.

### Statistical analysis

The sample size calculation was based on the number of patients (20,000) required to detect, with at least 95% confidence, serious adverse drug reactions (SADRs) including major hypoglycaemia events, with an incidence of 15 events 100,000 (0.015%) for a single modern insulin during the 6 months of the study. Assuming equal distribution of patients among the three modern insulins, the estimated sample size was 60,000 patients.

The influence of variables on vascular complications was evaluated using a univariate logistical regression model as a first step, run separately for macrovascular and microvascular complications. Patient and disease characteristics were included as variables in the model if, based on the literature, they were considered to be medically-related to vascular complications. These included: geographic region, age, sex, diabetes duration, BMI, HbA_1c_, FPG before breakfast, PPG after breakfast, TC, TG, creatinine (microvascular complications model only), HDL-C, LDL-C and SBP. Any significant correlates from this univariate logistical regression were subsequently entered into a stepwise multivariate logistical regression and only statistically significant correlates remained in the model. The statistical significance level employed was α = 0.05 (two tailed). P-values, odds ratio (OR) and 95% confidence intervals (CIs) were calculated.

## Results

### Baseline patient characteristics

Baseline patient characteristics are summarised in Table 1. Of the 66,726 patients enrolled in A_1_chieve: 17,806 had macrovascular complications and 38,670 had no macrovascular complications at baseline; 35,078 had microvascular complications and 21,190 had no microvascular complications at baseline; and 18,420 had no macrovascular or microvascular complications at baseline. South Asia had the youngest patients with macrovascular complications (mean 56.3 years) and Latin America the oldest (mean 65.1 years). The Middle East had the youngest patients with microvascular complications (mean 54.6 years) and Latin America the oldest (mean 61.0 years; Table 1). The highest BMI among patients with macrovascular complications was recorded in Russia and the lowest in China (Table 1). The same pattern was seen in patients with microvascular complications, with the highest in Russia and the lowest in China (Table 1). In patients with macrovascular complications, the longest mean diabetes duration was in Latin America and North Africa and the shortest in South Asia. Mean diabetes duration in patients with microvascular complications was longest in Latin America and North Africa and the shortest in South Asia (Table 1).

**Table 1:** Baseline patient characteristics by geographical region

	**All**	**China**	**South Asia**	**East Asia**	**North Africa**	**Middle East**	**Latin America**	**Russia**
*All patients*								
Age, yr								
n	65,381	10,889	21,775	9,966	4,011	14,549	1,126	3,065
mean (SD)	54.0 (12.0)	54.9 (14.7)	51.7 (10.2)	56.5 (12.1)	58.1 (11.8)	52.5 (11.4)	59.5 (12.9)	59.2 (9.2)
Sex								
n	66,656	11,019	22,436	10,030	4,032	14,927	1,138	3,074
male (%)	37,033 (55.6)	6,300 (57.2)	13,908 (62.0)	5,092 (50.8)	1,717 (42.6)	8,636 (57.9)	485 (42.6)	895 (29.1)
BMI, kg/m^2^								
n	59,115	10,817	19,292	8,664	3,611	12,571	1,092	3,068
mean (SD)	27.1 (5.0)	24.7 (3.4)	26.3 (3.8)	24.8 (4.3)	28.0 (5.0)	30.4 (5.5)	29.4 (5.6)	31.1 (5.3)
Diabetes duration, yr								
n	65,786	10,814	22,316	9,776	4,016	14,658	1,135	3,071
mean (SD)	8.0 (6.2)	6.3 (6.3)	6.4 (4.7)	8.6 (6.9)	11.5 (7.2)	9.8 (6.0)	12.0 (8.3)	8.4 (5.4)
*Patients with macrovascular complications*								
Age, yr								
n	17,506	2,318	4,806	2,662	972	4,195	333	2,220
mean (SD)	59.3 (10.6)	61.7 (14.1)	56.3 (8.9)	61.5 (10.6)	63.6 (9.5)	57.7 (10.1)	65.1 (9.9)	61.0 (8.5)
Sex								
n	17,786	2,342	4,943	2,684	978	4,278	335	2,226
male (%)	9,618 (54.1)	1,271 (54.3)	3,178 (64.3)	1,350 (50.3)	488 (49.9)	2,567 (60.0)	156 (46.6)	608 (27.3)
BMI, kg/m^2^								
n	16,271	2,306	4,645	2,310	867	3,603	317	2,223
mean (SD)	27.9 (5.3)	25.1 (3.4)	26.2 (3.6)	25.3 (4.3)	28.8 (5.1)	31.1 (5.5)	30.4 (6.0)	31.6 (5.3)
Diabetes duration, yr								
n	17,653	2,297	4,938	2,635	973	4,251	335	2,224
mean (SD)	10.3 (6.6)	9.4 (7.0)	8.4 (5.1)	10.5 (7.3)	14.2 (7.9)	12.4 (6.4)	14.6 (8.6)	9.2 (5.4)
*Patients with microvascular complications*								
Age, yr								
n	34,518	5,399	8,103	5,582	2,386	9,602	710	2,736
mean (SD)	56.9 (11.2)	57.9 (14.1)	54.8 (9.7)	59.0 (11.2)	60.9 (10.5)	54.6 (10.4)	61.0 (12.1)	59.8 (8.9)
Sex								
n	35,033	5,467	8,288	5,614	2,395	9,810	715	2,744
male (%)	18,542 (52.9)	2,957 (54.1)	5,067 (61.1)	2,752 (49.0)	994 (41.5)	5,703 (58.1)	293 (41.0)	776 (28.3)
BMI, kg/m^2^								
n	31,894	5,364	7,644	4,946	2,154	8,361	686	2,739
mean (SD)	27.6 (5.3)	24.7 (3.4)	26.1 (3.7)	25.0 (4.3)	28.4 (5.0)	30.9 (5.5)	29.4 (5.7)	31.2 (5.3)
Diabetes duration, yr								
n	34,666	5,371	8,281	5,514	2,379	9,667	712	2,742
mean (SD)	9.9 (6.4)	8.4 (6.7)	8.2 (4.9)	10.4 (7.2)	13.6 (7.3)	11.2 (6.0)	13.7 (8.3)	8.9 (5.4)

Note: due to the observational nature of this study, not all baseline data were recorded.

BMI = body mass index.

### Metabolic control and QoL by geographical region

Baseline HbA_1c_ and FPG values were similarly high across the different regions and in patients with macrovascular complications or microvascular complications (Table 2). Mean baseline HbA_1c_ values were ≥9.1% (≥76 mmol/mol) in patients with macrovascular complications across all regions and ≥9.4% (≥79 mmol/mol) across all regions in patients with microvascular complications. The baseline data for other measurements related to metabolic control and QoL are also displayed in Table 2.

**Table 2:** Baseline metabolic control and quality of life by geographical region

	**All**	**China**	**South Asia**	**East Asia**	**North Africa**	**Middle East**	**Latin America**	**Russia**
*Patients with macrovascular complications*								
HbA_1c_, %								
n	12,727	1,332	4,002	1,170	620	3,426	147	2,030
mean (SD)	9.5 (1.7)	9.1 (2.1)	9.3 (1.2)	9.7 (2.0)	9.6 (1.8)	9.7 (1.7)	9.6 (2.4)	9.6 (1.7)
HbA_1c_, mmol/mol								
n	12,727	1,332	4,002	1,170	620	3,426	147	2,030
mean (SD)	80.3 (18.6)	76.0 (23.0)	78.2 (13.1)	82.5 (21.9)	81.4 (19.7)	82.5 (18.6)	81.4 (26.2)	81.4 (18.6)
FPG before breakfast								
n	13,603	1,859	4,064	1,431	715	3,143	204	2,187
mmol/l, mean (SD)	10.7 (3.3)	9.8 (3.6)	10.8 (2.8)	11.2 (4.2)	11.2 (4.2)	11.0 (3.3)	10.7 (4.2)	10.4 (2.6)
mg/dl, mean (SD)	192.8 (59.6)	176.5 (64.0)	194.2 (50.5)	202.4 (74.9)	201.3 (75.7)	198.0 (60.1)	191.9 (76.5)	187.7 (46.9)
PPG after breakfast								
n	9,704	1,451	2,984	784	376	2,254	30	1,825
mmol/l, mean (SD)	14.5 (4.3)	14.0 (4.7)	15.3 (3.7)	15.4 (4.9)	14.7 (5.2)	15.4 (4.4)	13.1 (5.9)	12.2 (3.1)
mg/dl, mean (SD)	262.0 (77.0)	252.1 (85.4)	275.9 (65.9)	277.0 (87.6)	265.5 (94.0)	278.1 (78.9)	235.1 (105.6)	220.3 (55.6)
Total cholesterol, mmol/l								
n	7,176	851	466	744	378	2,530	136	2,071
mean (SD)	5.5 (1.3)	5.1 (1.2)	5.2 (0.8)	5.3 (1.5)	4.6 (1.3)	5.4 (1.2)	5.6 (1.7)	6.1 (1.3)
Triglycerides, mmol/l								
n	6,732	844	703	637	402	2,524	116	1,506
mean (SD)	2.1 (1.0)	2.0 (1.3)	2.2 (0.7)	2.0 (1.0)	1.6 (0.9)	2.3 (1.0)	2.4 (1.2)	2.1 (1.0)
Creatinine, μmol/l								
n	6,865	724	776	627	350	2,370	103	1,915
mean (SD)	87.7 (32.9)	48.7 (43.6)	96.2 (30.3)	97.6 (35.1)	89.5 (29.6)	95.7 (29.4)	94.5 (28.6)	85.2 (19.5)
HDL-C, mmol/l								
n	5,602	777	714	539	255	2,283	98	936
mean (SD)	1.1 (0.4)	1.2 (0.4)	1.0 (0.2)	1.2 (0.5)	1.1 (0.4)	1.0 (0.3)	1.0 (0.3)	1.4 (0.6)
LDL-C, mmol/l								
n	5,711	794	713	545	238	2,364	94	963
mean (SD)	3.2 (1.1)	3.0 (1.0)	3.4 (1.0)	3.4 (1.3)	2.8 (1.4)	3.2 (1.0)	3.2 (1.1)	3.4 (1.2)
SBP, mmHg								
n	13,744	1,552	3,376	1,969	755	3,619	274	2,199
mean (SD)	141.0 (19.5)	137.2 (18.3)	145.2 (21.0)	133.3 (18.7)	137.8 (20.0)	141.4 (18.9)	131.8 (16.5)	145.7 (16.5)
QoL*								
n	11,329	1,712	3,611	2,156	676	865	261	2,048
mean (SD)	60.2 (16.5)	74.4 (13.9)	52.7 (10.6)	66.7 (15.7)	59.4 (17.8)	62.1 (14.9)	63.7 (17.8)	53.5 (16.9)
*Patients with microvascular complications*								
HbA_1c_, %								
n	24,352	3,128	6,673	2,385	1,545	7,726	378	2,517
mean (SD)	9.6 (1.7)	9.4 (2.3)	9.4 (1.3)	9.7 (2.0)	9.5 (1.8)	9.7 (1.7)	10.1 (2.2)	9.6 (1.7)
HbA_1c_, mmol/mol								
n	24,352	3,128	6,673	2,385	1,545	7,726	378	2,517
mean (SD)	81.4 (18.6)	79.2 (25.1)	79.2 (14.2)	82.5 (21.9)	80.3 (19.7)	82.5 (18.6)	86.9 (24.0)	81.4 (18.6)
FPG before breakfast								
n	25,986	4,239	6,655	3,015	1,744	7,162	480	2,691
mmol/l, mean (SD)	10.8 (3.5)	10.0 (3.5)	10.7 (3.0)	11.3 (4.2)	11.1 (4.1)	11.2 (3.5)	11.7 (4.6)	10.4 (2.7)
mg/dl, mean (SD)	194.5 (62.9)	179.4 (63.6)	193.3 (54.3)	202.8 (75.5)	200.4 (74.5)	201.5 (62.4)	210.5 (83.3)	186.9 (47.7)
PPG after breakfast								
n	18,300	3,297	4,762	1,657	1,025	5,183	110	2,266
mmol/l, mean (SD)	14.8 (4.5)	13.8 (4.8)	15.5 (3.9)	15.6 (5.0)	14.8 (4.7)	15.5 (4.4)	15.6 (6.0)	12.1 (3.0)
mg/dl, mean (SD)	265.8 (80.3)	249.4 (86.7)	279.0 (69.7)	281.5 (89.3)	265.7 (84.6)	279.6 (80.1)	281.4 (107.3)	218.3 (54.9)
Total cholesterol, mmol/l								
n	13,795	2,005	812	1,509	993	5,590	334	2,552
mean (SD)	5.4 (1.3)	5.1 (1.3)	5.1 (0.8)	5.3 (1.4)	4.7 (1.3)	5.3 (1.2)	5.8 (1.6)	6.0 (1.3)
Triglycerides, mmol/l								
n	13,356	1,970	1,371	1,257	1,030	5,572	290	1,866
mean (SD)	2.1 (1.1)	2.1 (1.3)	2.1 (0.8)	2.0 (1.1)	1.6 (0.9)	2.2 (1.0)	2.5 (1.3)	2.1 (1.0)
Creatinine, μmol/l								
n	13,399	1,779	1,482	1,185	893	5,440	261	2,359
mean (SD)	83.5 (32.7)	52.3 (42.3)	89.9 (27.9)	95.8 (37.9)	85.0 (28.2)	88.2 (28.6)	89.2 (26.4)	84.8 (19.1)
HDL-C, mmol/l								
n	11,472	1,823	1,398	1,083	678	5,067	234	1,189
mean (SD)	1.1 (0.4)	1.2 (0.4)	1.0 (0.2)	1.2 (0.4)	1.1 (0.4)	1.1 (0.3)	1.0 (0.3)	1.4 (0.6)
LDL-C, mmol/l								
n	11,580	1,852	1,379	1,090	646	5,185	217	1,211
mean (SD)	3.2 (1.1)	3.1 (1.1)	3.1 (0.9)	3.2 (1.2)	2.9 (1.4)	3.2 (1.0)	3.2 (1.1)	3.3 (1.1)
SBP, mmHg								
n	26,661	3,487	5,656	4,022	1,899	8,282	614	2,701
mean (SD)	137.3 (18.7)	134.9 (17.5)	141.4 (21.0)	132.0 (17.9)	135.2 (18.8)	137.1 (17.4)	131.5 (17.2)	143.1 (16.8)
QoL*								
n	21,166	3,947	6,103	4,489	1,741	1,805	555	2,526
mean (SD)	62.3 (16.9)	75.2 (13.8)	52.8 (11.1)	67.9 (16.0)	61.1 (17.5)	64.1 (15.5)	64.8 (18.3)	54.3 (17.0)

Note: due to the observational nature of this study, not all baseline data were recorded.

*EQ-5D™ (100 = best imaginable health; 0 = worst imaginable health) [20].

### Complications by geographical region

There were 17,806 patients with reported macrovascular complications and 35,078 with microvascular complications (Table 3). Macrovascular complications were reported in 27% of participants overall, with Russia having the highest percentage (72%) and China the lowest (21%) (Table 3). Microvascular complications were reported in 37 to 89% of participants, with Russia having the highest percentage and South Asia the lowest. Neuropathy was the highest reported microvascular complication in all regions, ranging from 25% in South Asia to 83% in Russia.

**Table 3:** Baseline patient complications by geographical region

	**All**	**China**	**South Asia**	**East Asia**	**North Africa**	**Middle East**	**Latin America**	**Russia**
*Patients with macrovascular complications, n (%)*	17,806 (27.2)	2,342 (21.3)	4,946 (23.3)	2,685 (26.8)	979 (24.2)	4,293 (28.7)	335 (29.4)	2,226 (72.4)
*Patients with microvascular complications, n (%)*	35,078 (53.5)	5,467 (49.6)	8,293 (39.0)	5,615 (56.0)	2,397 (59.4)	9,847 (65.8)	715 (62.8)	2,744 (89.3)
Renal disease, n (%)	18,271 (27.9)	2,455 (22.3)	4,321 (20.3)	2,845 (28.4)	1,077 (26.7)	6,108 (40.8)	376 (33.0)	1,089 (35.4)
Eye problems, n (%)	17,198 (26.3)	2,430 (22.1)	3,464 (16.3)	2,380 (23.7)	1,354 (33.5)	5,081 (33.9)	368 (32.3)	2,121 (69.0)
Foot ulcer, n (%)	3,538 (5.4)	274 (2.5)	1,046 (4.9)	536 (5.3)	147 (3.6)	1,289 (8.6)	85 (7.5)	161 (5.2)
Neuropathy, n (%)	25,179 (38.4)	3,671 (33.3)	5,234 (24.6)	3,706 (36.9)	1,530 (37.9)	7,995 (53.4)	491 (43.1)	2,552 (83.0)

Note: due to the observational nature of this study, not all baseline data were recorded.

### Regression analysis

**Figure 1:**
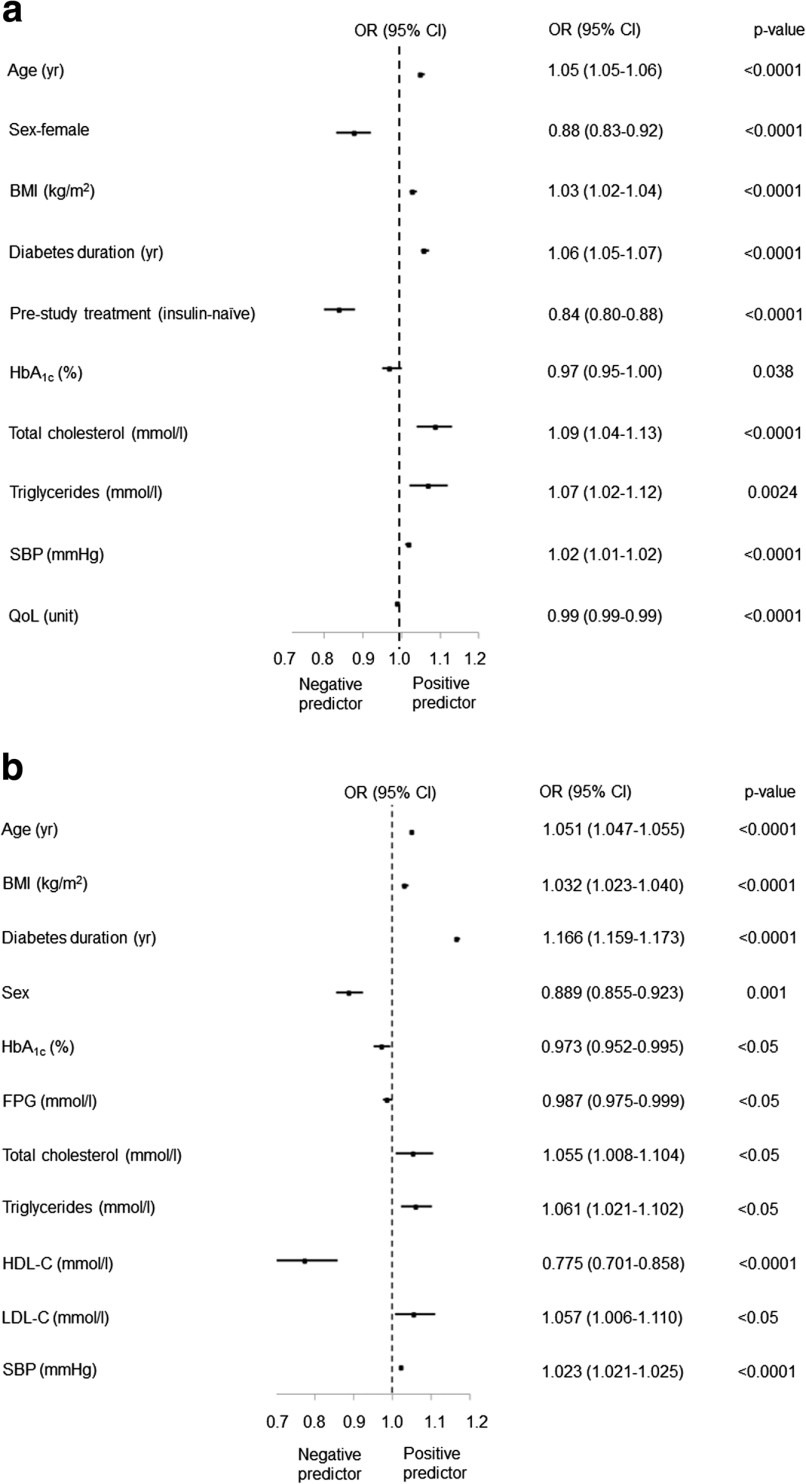
**Complication predictor analysis. a)** Macrovascular complications. **b)** Microvascular complications. OR = odds ratio; CI = confidence interval.

The univariate logistical regression revealed that all correlates entered into both models were statistically significant (all p < 0.05). Subsequently, all correlates were entered into a stepwise multivariate logistical regression, and only statistically significant association remained in the models. After adjusting for regional differences, age, BMI, diabetes duration, TC, TG, LDL-C and SBP were positively correlated with macrovascular complications (all p < 0.05; Figure 1a). Female gender, HDL-C, FPG and HbA_1c_ were negatively correlated with macrovascular complications (all p < 0.05; Figure 1a). Age, BMI, diabetes duration, HbA_1c_, LDL-C, creatinine, and SBP were positively correlated with microvascular complications (all p < 0.01; Figure 1b). HDL-C levels were negatively correlated with microvascular complications (p < 0.001; Figure 1b).

### Use of vascular disease preventative drugs by geographical region

Among participants with macrovascular complications overall, 67% reported taking statins, while the proportion was 56% among participants with microvascular complications. For participants with macrovascular complications and those with microvascular complications, the highest statin use was in the Middle East (86% and 74%, respectively) and the lowest was in China (49% and 34%, respectively). Use of vascular disease preventative drugs was higher among participants with macrovascular complications (statins 67%, aspirin 79%, RAS blocker 78%) than those with microvascular complications (statins 56%, aspirin 64%, RAS blocker 63%) (Table 4).

**Table 4:** Use of vascular disease preventative drugs by geographical region

	**All**	**China**	**South Asia**	**East Asia**	**North Africa**	**Middle East**	**Latin America**	**Russia**
*Patients with macrovascular complications*								
RAS blocker, n (%)	13,910 (78.1)	1,303 (55.6)	3,771 (76.3)	1,992 (74.2)	778 (79.5)	3,719 (86.6)	285 (85.1)	2,062 (92.6)
Aspirin, n (%)	13,995 (78.6)	1,644 (70.2)	3,784 (76.6)	1,866 (69.5)	801 (81.8)	3,973 (92.5)	252 (75.2)	1,675 (75.2)
Statins, n (%)	11,958 (67.2)	1,139 (48.6)	3,057 (61.9)	1,846 (68.8)	715 (73.0)	3,710 (86.4)	189 (56.4)	1,302 (58.5)
*Patients with microvascular complications*								
RAS blocker, n (%)	21,884 (62.6)	2,268 (41.5)	4,792 (58.5)	3,465 (61.7)	1,557 (65.0)	6,907 (70.1)	499 (69.8)	2,396 (87.3)
Aspirin, n (%)	22,207 (63.5)	2,704 (49.5)	4,958 (60.6)	2,698 (48.0)	1,598 (66.7)	7,922 (80.5)	427 (59.7)	1,900 (69.2)
Statins, n (%)	19,709 (56.4)	1,856 (33.9)	4,325 (52.8)	3,133 (55.8)	1,288 (53.7)	7,323 (74.4)	312 (43.6)	1,472 (53.6)

Note: due to the observational nature of this study, not all baseline data were recorded.

RAS = renin-angiotensin system.

In all regions, aspirin use was higher in participants with macrovascular complications than those with microvascular complications (Table 4). In addition, aspirin use was higher than statin use in all regions except East Asia, where usage levels of the two drugs were similar among participants with macrovascular complications, and statin use was higher than aspirin use among participants with microvascular complications (Table 4).

A higher proportion of participants with macrovascular complications used a RAS blocker than those with microvascular complications in all regions apart from Russia, where usage was similar for the two groups. Russia also had the highest use of RAS blocker, both among participants with macrovascular complications (93%) and those with microvascular complications (87%). In contrast, use of RAS blocker was lowest in China for both participant groups (56% of participants with macrovascular complications and 42% of participants with microvascular complications).

## Discussion

Baseline HbA_1c_ was high across all geographical regions, and vascular complication rates were generally high. In addition, the use of vascular protective therapies, even in those patients with vascular complications, was sub-optimal. The particularly low reported use of vascular protective therapies in patients from China is commensurate with previous findings [21]. The findings suggest poor glycaemic control and suboptimal diabetes management across many geographical regions, which may be due to poor adherence to treatment regimens, lack of access to therapy, poor diet, and delay in initiating, or failure to adequately optimise, insulin therapy [12-14,16,22]. For example, significant numbers of people with diabetes have poor psychological well-being and these psychological problems can adversely affect adherence to treatment [22]. Some of the variation seen in the prevalence of complications in different geographical regions may have been due to variations in clinical care, healthcare resources, diagnostic criteria, and definitions used in routine clinical practice, among other reasons. In particular, the high rates of complications among participants from Russia may have been due to a variety of factors, such as late diagnoses of diabetes, late initiation of treatment, or lifestyle factors. Without large-scale epidemiological studies, it is difficult to ascertain the extent to which clinical practice may impact on the rate of complications in people with diabetes in Russia or other regions.

Results of the correlation analysis revealed relationships between vascular complications and various disease characteristics. Age, BMI, diabetes duration, LDL-C, and SBP were positively associated with macrovascular and microvascular complications, while TC and TG were positively correlated with macrovascular complications and creatinine with microvascular complications. These findings are consistent with the large evidence base showing that the risk of developing these complications is positively associated with these variables [23-27]. The negative association between HDL-C values and macrovascular and microvascular complications would also be expected given the documented inverse relationship between blood levels of HDL-C and cardiovascular disease risk [28]. The profile of cholesterol levels is important in people with type 2 diabetes as it reflects atherogenic dyslipidaemia, which is characterised by small dense LDL-C, low HDL-C and high TG levels [29]. Regarding the result suggesting female gender was associated with a lower frequency of macrovascular complications, past research in this area has shown mixed results [30-32].

In contrast, the finding that HbA_1c_ and FPG values were negatively associated with establishment of macrovascular complications was surprising. The correlation coefficient between HbA_1c_ and FPG values was r = 0.44, and therefore, colineairty is not likely to have distorted the model and explain this finding. One possible explanation for this surprising result is the study design. The A_1_chieve study was a cross-sectional study and as such, the present baseline HbA_1c_ and FPG values were used to predict the establishment of diabetes complications which had developed over the course of the disease. However, there may not be a clear association between the present value and the value several years prior to this. In addition, complications (especially macrovascular complications) usually take several years to develop in the presence of high HbA_1c_ levels, and so an association would become apparent in a cohort study rather than a cross-sectional study. The temporal relationships between HbA_1c_ levels and establishment of macrovascular and/or microvascular complications must be further established before fully understanding the power of current HbA_1c_ levels to predict complications.

Alternative explanations for this unusual finding could be the fact that the study population included a very heterogenous group ranging across four continents, leading to genetic variability, dietary variability and risk factor variability. Minimising these confounding factors may have provided a clearer picture of the relationships between the various disease parameters and vascular complications and this could be an area for future research. In addition, there were no specific or defined measurements of the macrovascular and microvascular complications reported in this study, and these were classified based on clinical judgement of the physicians. Other limitations of this observational study include potential bias and lack of a control group; however, an advantage of this observational study was that it had less stringent inclusion and exclusion criteria, allowing very large numbers of people to be assessed in many different geographical regions, something that is virtually impossible with randomised controlled trials.

## Conclusions

Across all the geographical regions, patients showed poor glycaemic control at the point of commencing on insulin analogues, vascular complication rates were generally high, and use of vascular disease preventative therapies was generally sub-optimal. Many of the disease characteristics were statistically significantly associated with macrovascular and/or microvascular complications at baseline. These findings suggest that suboptimal diabetes therapy is contributing to the burden of type 2 diabetes worldwide. Closer adherence to diabetes guidelines, which may include earlier initiation and optimisation of insulin regimens (e.g., with insulin analogues in the A_1_chieve population), may reduce the prevalence of vascular complications, improve the lives of people with diabetes and reduce the burden on healthcare systems.

### Consent

Participants gave written informed consent for the results of the study to be published in a scientific or medical journal.

## Abbreviations

ADA: American Diabetes Association; BMI: Body mass index; CIs: 95% confidence intervals; EASD: European Association for the Study of Diabetes; EQ-5D: European questionnaire – 5 dimensions; FPG: Fasting plasma glucose; HbA1c: Glycated haemoglobin; HDL-C: High-density lipoprotein-cholesterol; IDF: International Diabetes Federation; LDL-C: Low-density lipoprotein-cholesterol; mg/dl: Milligram per decilitre; mm/Hg: Millimetres of mercury; mmol/l: Millimoles per litre; MI: Myocardial infarction; n: Number; OR: Odds ratio; OGLDs: Oral glucose-lowering drugs; P: Probability; PPG: Post-prandial plasma glucose; QoL: Quality of life; RAS: Renin-angiotensin system; SADRs: Serious adverse drug reactions; SD: Standard deviation; SBP: Systolic blood pressure; TC: Total cholesterol; TG: Triglycerides; UKPDS: United Kingdom Prospective Diabetes Study.

## Competing interests

LL is a speaker and member of the Latin American Board of Eli Lilly, and the Argentinean Boards of Novo Nordisk, Novartis, Sanofi, BMS, and Astra Zeneca. He is also a principal investigator of clinical trials run by Eli Lilly, Novo Nordisk, and Astra Zeneca. SYG and her affiliated institution have received funding from Novo Nordisk and Sanofi for research, advisory and educational activities. ZH has received funding from Novo Nordisk, Eli Lilly and Sanofi for research, advisory and education activities. RM has participated in several advisory boards for Novo Nordisk and has participated in studies sponsored by Novo Nordisk. He has also participated in symposia run by Novo Nordisk, Sanofi, Eli Lilly, Merck, and Pfizer. VP is an employee of Novo Nordisk A/S. MEK is a principal investigator of a clinical trial in Iran run by Novo Nordisk A/S and also an observational study supported by Sanofi.

## Authors’ contributions

LL, SYG, ZH, RM, VP and MEK were all involved in the interpretation of data, involved in drafting the manuscript or revising it critically for important intellectual content and have given final approval of the version of the manuscript to be published.
